# Patient-Related Barriers to Chronic Pain Management Among Cancer Survivors: A Narrative Synthesis

**DOI:** 10.21203/rs.3.rs-6296813/v1

**Published:** 2025-04-04

**Authors:** Jonathan Zhu, Connie Ulrich, Peggy Compton, Jie Deng

## Abstract

**Purpose** : Cancer survivors often experience chronic pain, impacting their daily functioning and quality of life. Despite the existence of effective pain management therapies, cancer survivors may not experience adequate pain management related to patient-related barriers. This review aims to explore the patient-related barriers that inhibit effective chronic pain management among cancer survivors. **Methods** : A narrative synthesis was conducted using a systematic review of the literature. Abstract and full-text screening, along with quality appraisal and level of evidence assessments, were conducted. Data was extracted on each study’s approach, methods, results, and implications, and thematic analysis was used to synthesize the findings. **Results** : A total of 330 participants were included in the 11 papers reviewed. General barriers to chronic pain management included a lack of preparedness for chronic pain, negative perceptions toward chronic pain, and poor patient-provider communication. Opioid-specific barriers were identified and included opioid use disorder stigma, knowledge deficits about opioid-related terms, and undesirable side effects. Physical activity barriers included logistical considerations, symptom-related restrictions, and cognitive challenges. **Conclusions** : Patient-related barriers to chronic pain management among cancer survivors include general, opioid-related, and physical activity-related barriers. Among these, the most significant barriers were negative perceptions toward chronic pain, inadequate patient-provider communication, opioid-related stigma, undesirable side effects of opioids, and symptom-related barriers to physical activity. **Implications** : Healthcare providers, cancer survivors, and caregivers require more education on communication techniques, the biopsychosocial model of pain, and opioid use disorder. Social support interventions could also be developed to promote engagement in physical activity among cancer survivors.

## Background

As patients with cancer experience prolonged survival due to the advent of novel treatments and technologies [1–4], chronic cancer pain among cancer survivors has become an increasingly problematic outcome. Recent studies estimate that over 30% of cancer survivors experience chronic pain after primary curative treatment, with that number rising to 66% among survivors with advanced and metastatic cancer [5, 6]. The negative effects of chronic pain, defined as recurrent pain that lasts beyond 3 months [7], are extensive, including restricted ability to perform daily activities, decreased self-efficacy, inhibited socialization, and diminished overall quality of life [8, 9]. The etiology of chronic cancer pain is often complicated and multifaceted, and can include complex pain syndromes, lymphedema-related pain, and treatment-related factors such as surgery, radiation therapy, or chemotherapy-induced peripheral neuropathy [9, 10].

Chronic cancer pain is primarily treated with pharmacotherapy, which includes opioid, non-opioid, and adjuvant analgesics [9, 11]. Medications for chronic cancer pain are prescribed according to the World Health Organization Analgesic Ladder Model, which begins with non-opioids and adjuvants for minimal pain levels, and progresses to opioids for mild to severe pain [12]. In practice, opioid analgesics are most commonly prescribed for chronic cancer pain management and are highly effective, producing therapeutic pain relief for 95% of patients with moderate to severe cancer pain [13]. Non-opioid and adjuvant analgesics used for chronic cancer pain include acetaminophen, ibuprofen, clonidine, ketamine, antidepressants, and gabapentin [13]. Studies on the efficacy of these medications for managing chronic cancer pain have yielded mixed results, with a few reporting minimal or insignificant pain relief [14, 15].

Non-pharmacological and non-traditional medicine approaches are also recommended for chronic cancer pain management. Engaging in routine physical activity has been shown to not only reduce pain, but to also improve physical functioning, fatigue, and overall quality of life [16, 17]. In a set of published guidelines on nutrition and physical activity for cancer survivors, the American Cancer Society recommends that cancer survivors aim to exercise for at least 150 minutes per week [18]. In the past two decades, providers have also increasingly focused on incorporating multimodal, complementary interventions for chronic cancer pain [11, 19]. These include techniques such as heat/cold therapy, eastern medicine therapy, cannabis, massage, acupuncture, hypnotherapy, aromatherapy, music therapy, transcutaneous electric nerve stimulation, and cognitive behavioral therapy [20, 21].

Despite the efficacy of many of these therapies, chronic cancer pain is too often poorly managed, with studies estimating that 30–50% of patients with chronic cancer pain are undertreated [22, 23]. Barriers to adequate chronic cancer pain management include clinician-related factors, patient-related factors, systemic and regulatory obstacles, societal attitudes toward pain management, and racial and socioeconomic disparities in pain assessment and management [24]. Among these, there is a need for research focused on patient-related barriers, as developing a more comprehensive understanding of cancer survivors' experiences with chronic pain management will most effectively inform the development or implementation of effective interventions [25, 26]. Previous literature has identified patient-related barriers such as lack of provider access in rural areas [27], high out-of-pocket costs due to a lack of insurance coverage [9], and the burden of managing multiple comorbidities [27]. To build on these findings, the purpose of this narrative review is to explore patient-related barriers to effective pain management among cancer survivors. The narrative synthesis is a systematic approach that aims to "tell the story" for research questions with a broad range of study designs, which was appropriate for this review of both qualitative and quantitative studies [28–30].

## Methods

This review was conducted in accordance with the Preferred Reporting Items for Systematic reviews and Meta-Analyses (PRISMA) reporting guidelines [31] (see Appendix A), which is appropriate for systematic reviews of quantitative and qualitative studies, as well as the Synthesis Without Meta-analysis (SWiM) reporting guideline from the Equator Network [32], which is intended to be used as an extension to PRISMA guidelines when conducting narrative syntheses (see Appendix B).

### Literature Search

A systematic search of the literature was conducted during June and July of 2024 with the assistance of a University of Pennsylvania biomedical librarian. PubMed and CINAHL were searched for this review due to their comprehensive coverage of biomedical, nursing, life science, and healthcare-related articles, which align closely with the population of this review. Scopus and Embase searches were incorporated for their broader scope across various scientific disciplines, allowing for a more thorough examination of the pain management literature. Covidence was utilized to optimize the screening process and provide a more efficient approach to data management [33, 34].

Search terms used are provided in Appendix C, yielding a total of 172 articles after duplicates were automatically removed (see Appendix D). Articles were screened based on the following inclusion criteria: Participants at least 18 years of age; participants completed intensive curative cancer treatment; and mentions patient-related barriers to chronic cancer pain management. Articles were excluded if any of the exclusion criteria were met: Participants undergoing active acute cancer treatment; focused on quality-of-life outcomes, risk factors for pain, or non-cancer pain. While numerous definitions of cancer survivorship have been purported [35], this search focused on cancer patients who have completed primary, intensive curative treatment (ex: surgery, chemotherapy, radiation therapy) with managed chronic or intermittent disease [2, 36], which is the most commonly utilized definition among health professionals [37].

Screening was conducted by two members of the review team (JZ, PC), where articles were first reviewed independently (initial agreement: 97%), and discrepancies were then collectively discussed. The first stage of screening involved reviewing article abstracts, which produced 39 articles for full-text review. During full-text review, 28 articles were excluded for the following reasons: non-systematic review papers (10), incomplete studies (9), different patient populations (4), informal study designs (3), irrelevant outcomes (1), or duplicate publications (1). Following this screening process, 11 articles were included in the final review (8 qualitative research articles, 2 systematic reviews, and 1 quantitative research article).

#### Quality Appraisal of Studies

The Joanna Briggs Institute's (JBI) Critical Appraisal Tools were used to conduct quality assessments on all of the included papers [38]. The JBI tool was utilized because it provides specific appraisal checklists and guides for different types of study designs, thus for this review, the qualitative research [39], systematic reviews and research syntheses [40], and analytical cross sectional studies JBI tools [41] were used (See Appendix E). Quality appraisal was conducted by two members of the study team independently (JZ, PC); initial agreement was 92%, and discrepancies were discussed to reach a consensus.

Two articles were appraised as being high-quality evidence (10/11 criteria met)[42, 43]; the remaining nine articles were scored as being of moderate-quality evidence (6/8, 6/10 or 7/10 criteria met)[9, 44-51]. Among the 8 qualitative research articles, the quality rating of all were related to not stating the underpinning philosophical perspective, not identifying the researcher's cultural or theoretical location, or not addressing the influence of the researcher on the research. Neither of the two systematic review articles [42, 43] assessed the likelihood of publication bias. The analytical cross-sectional study did not use an objective standard for the measured condition or measure the outcomes in a reliable manner [52].

#### Level of Evidence

The level of evidence of each included paper was also assessed to evaluate the research design, quality of the study, and applicability to patient care. Using the classification system from *Evidence-based practice in nursing & healthcare: A guide to best practice* [53], "Level 1" represents the highest quality of evidence with the lowest risk of bias, while "Level 7" is the lowest score an article can receive. Of the papers included, one article was graded as "Level 4," which reflects evidence from a well-constructed cohort study [52]. Two articles received a "Level 5" score, which represents evidence from systematic reviews of qualitative studies [42, 43]. The remaining 8 articles were graded as "Level 6," which is evidence from a single qualitative study [9, 44-50]. A table summarizing the quality appraisal and level of evidence of each paper is included (see Table 1).

#### Data Extraction and Analysis

In the preliminary synthesis, the findings from the included studies were synthesized using thematic analysis [54], which is a recommended tool for conducting a narrative synthesis [28]. Thematic analysis consists of 6 main steps: becoming familiar with the data; generating initial codes; searching for themes; reviewing themes; defining themes; and writing up the synthesized results [54]. For this review, the process began with data extraction, where information regarding the research approach, study design, research objectives, sample, data collection, data analysis, results (or findings), and discussion (or conclusions, implications, and/or significance) of each study were extracted. As none of the qualitative studies mentioned specific study designs, information from the methods sections were used to determine the appropriate qualitative study designation. These findings were summarized in a table of evidence (see Table 2). Next, the extracted data was reviewed to develop an initial understanding of the findings. An initial set of codes were then generated using open coding, which was developed and adapted throughout the coding process [55]. Following multiple comprehensive readings of the studies, emergent themes were identified, evaluated for consistency and accuracy, and defined.

The complete coding process, including initial codes, themes, sub-themes, descriptions, and examples, was completed by hand by the first author and is presented in the thematic analysis table (see Table 3). Prominent recurring ideas identified through this process were (1) lack of preparedness for chronic pain; (2) negative perceptions toward chronic pain; (3) patient-provider communication; (4) opioid-related stigma; (5) opioid-related knowledge deficits; (6) undesirable side effects of opioids; (7) logistical barriers to physical activity; (8) symptom-related barriers to physical activity; (9) cognitive barriers to physical activity; and (10) financial barriers to alternative pain management modalities.

## Results

Following coding, the resultant narrative synthesis emerged. Building upon the thematic analysis described above, an exploration of the relationships within and between study findings was conducted (concept mapping) [28].

**I. Preliminary Synthesis (Thematic Analysis)**

**Sample Demographics**

A total of 330 participants were involved in the included studies. Most (n = 6) included participants from the United States [9, 43-45, 47, 50] while the remaining five included participants from European and Asian countries (France, Italy, Norway, and Singapore) [42, 46, 48, 49, 52]. Most participants (86.4%) were female and among studies reporting median ages, the range was 54.5-64.4 years, while the mean age across studies reporting means was 56.9 years. Of the studies that reported race/ethnicity, most of the participants included were White (53%), although a few studies focused specifically on Black participants [9, 44, 50].

Ten of the articles included cancer survivors diagnosed with breast cancer [9, 42-44, 46-50, 52], and the other represented various types of cancer survivors (i.e., hematologic, head and neck, prostate, colorectal, gastrointestinal, reproductive, hepatobiliary and lung) [45, 47, 51, 52]. The participants varied in their cancer survivorship trajectories, ranging from 7 months post-intensive treatment to 10 years post-treatment. One study included patients who had completed a randomized controlled trial focusing on a behavioral intervention for cancer-related chronic pain [47].

**Types of Chronic Cancer Pain Management**

*Non-Opioid Analgesics*

Non-opioid analgesics were mentioned as a common treatment option for pain management across several studies [9, 45, 48, 50]. These medications included acetaminophen [9, 45, 48, 50], ibuprofen [9, 48, 50], antidepressants [9, 45], and gabapentin [9, 45]. However, across these studies, non-opioid analgesics were found to have a minimal effect on alleviating chronic pain for many cancer survivors [9, 48, 50]. As a result, some participants gradually stopped taking non-opioid analgesics and expressed a desire for more effective pain management options [9, 48].

*Opioid Analgesic Therapy*

Opioid analgesic therapy was the most common modality for cancer-related pain management, as it was mentioned in 5 of the articles [9, 44, 45, 49, 50]. Prescription opioid medications included oxycodone and hydrocodone and were usually utilized after nonopioid analgesics were proven to be less than effective alone [9, 44, 50].

*Physical Activity*

Physical activity approaches to cancer-related pain management were also mentioned in several studies [9, 43, 45, 47-49, 52]. The different forms of physical activity mentioned in the articles included walking [43, 47, 49], swimming [49], yoga [43, 49], range of motion or stretching exercises [9], and Qigong [43]. One study found that the majority of cancer survivors (75.7%) met the American Cancer Society's recommendation of 150 minutes of moderate or 75 minutes of vigorous aerobic exercise per week [52].

*Alternative Pain Management Modalities*

Some studies mentioned alternative modalities for cancer-related pain management [9, 44, 45]. Cannabis was mentioned as an alternative pharmacological pain management intervention [44, 45]. Non-pharmacological alternatives included acupuncture [44, 45], herbs [9, 45], vitamin supplements [9], essential oils [9], massages [44, 45], hot/cold compresses [9], warm baths [9], aromatherapy [9], and meditation [9, 45].

**General Barriers to Effective Pain Management**

With respect to patient-related barriers to effective chronic cancer pain management, some were identified as more general barriers, whereas others were barriers more specific to opioid therapy, physical activity, and alternative pain management modalities.

*Lack of Preparedness for Chronic Pain*

Of the general barriers, cancer survivors expressed that they were not properly informed about the risk of developing chronic pain following their cancer treatment [42, 48]. Participants mentioned that they were not educated on what a "normal" level of post-cancer pain would be [48]. In many cases, even if the provider did mention the possibility of developing chronic pain, it was done during the acute phase of treatment when patients were more focused on cancer prognosis and surgery [42, 48]. Patients reflected on a desire to be reminded of the chronic pain prognosis throughout the cancer recovery process. Due to these gaps in care, in one study, participants mentioned experiencing a disconnect between their expectations of recovery and their lived experiences of chronic pain, which led to feelings of frustration and loneliness [42].

*Negative Perceptions Toward Chronic Pain*

Cancer survivors demonstrated a variety of negative perceptions toward chronic pain that affected their approaches to pain management [42, 46-49]. Some participants adopted a fatalistic view of chronic pain, viewing it as an unavoidable condition that would never go away [42, 46]. This commonly resulted in stoic responses where they felt they needed to learn to live with their pain and endure even high levels of pain because they were unsure if it was severe enough to seek treatment [42, 48].

The combination of fatalistic and stoic outlooks contributed to a widespread minimization of pain [47, 49]. Some participants described feeling like they needed to downplay their pain because they should feel lucky "just to be alive" [47]. These findings were also reflected by discrepancies between quantitative survey responses and qualitative interview responses, where some participants described low levels of pain, but upon further questioning, explained that it severely inhibited their ability to perform daily tasks [46].

*Patient-Provider Communication*

There were several communication barriers to chronic cancer pain management between cancer survivors and healthcare providers [9, 42, 44, 46-49]. Three studies mentioned communication barriers with doctors [42, 47, 49], and four articles mentioned communication barriers with both nurses and doctors [9, 44, 46, 48]. Some cancer survivors reported having difficulty verbally relaying their experiences to their providers [42, 47]. They conveyed feelings of frustration as they tried to describe their physical sensations, often resorting to metaphors rather than explicitly naming their sensations as pain [42].

Participants also perceived a lack of empathy and understanding from providers [9, 42, 46, 47, 49]. They felt like their pain was misunderstood because providers would downplay their level of pain or interpret it as only physical [46, 49]. In other instances, providers made immediate referrals to psychiatrists without seeking to properly understand their patients' concerns [42]. These interactions left patients feeling frustrated and guilty because it questioned the legitimacy of their pain [42, 46, 49]. Some cancer survivors expressed that providers would give very little instructions about their opioid medications and only ask short, close-ended questions that made it difficult to share about their pain experiences [44]. Participants described wanting to avoid being perceived by providers as "complaining," "being difficult," or "drug-seeking" [9, 47]. When patients felt that they could not trust their providers because of a lack of empathy, they were more likely to engage in detrimental self-directed practices such as abruptly halting their opioid use [44], as well as avoid seeking assistance with pain management [47].

Importantly, cancer survivors described uncertainty about which healthcare providers were primarily responsible for managing their pain [9, 47]. Very few participants were able to point to a specific clinician as their primary provider for pain management [47]. This confusion resulted in delayed care, where patients would have to wait several weeks before finally being able to schedule an appointment to discuss their ongoing pain [9].

**Barriers Specific to Opioid Analgesic Therapy**

*Opioid-Related Stigma*

Among cancer survivors, fear of opioid-related harms such as physical dependence, use disorder, and overdose were common [9, 44, 45, 48, 50]. Participants described opioids using stigmatizing terms such as "hard drugs," "illegal," "addictive," "street drug," "overdose," and "habit-forming" [50]. These fears were exacerbated by negative perceptions of opioid use in the media (ex: news and internet), close social circles (experiences of friends and family), or personal experiences with opioid use [50]. Family and friends sometimes played an active role in discouraging or withholding opioid-use for pain management [44].

When asked about which opioid medications they were most familiar with, participants mentioned heroin, fentanyl, and morphine, despite oxycodone and hydrocodone being the most commonly prescribed opioid medications [50]. Fears described above compelled some cancer survivors to either take lower dosages of opioids than prescribed (just enough to "take the edge off [of pain]") [9], or avoid opioid analgesic therapy altogether [44]. Participants also conveyed that they would only consider taking opioids if they had no other options for severe pain [45]. These various sources of stigma related to opioid use resulted in suboptimal pain management [44], which was sometimes described as preferable by participants over risking the possibility of developing a use disorder [9].

*Opioid-Related Knowledge Deficits*

Cancer survivors expressed several concerns regarding their lack of knowledge in opioid use [44, 50]. Participants reported feeling that they were not sufficiently educated on proper opioid use, storage, and disposal [44]. They described seeking information about proper disposal from multiple sources, such as the hospital, fire station and their pain medicine doctors, but not receiving any helpful directions [44].

In addition, there were knowledge deficits in participants' understandings of terms associated with opioid use and use disorders [44, 50]. Cancer survivors incorrectly described or interchanged terms such as dependence, tolerance, withdrawal, addiction, and pseudo-addiction [44, 50]. Participants also adopted incorrect perceptions of how opioid use disorder develops, such as believing that users become "addicted" immediately, and that opioids are misused because the pain level exceeds the prescribed dosage [50]. Inaccurate understanding of the development of a use disorder resulted in reluctance to take prescribed opioid medications [44, 50].

*Undesirable Side Effects of Opioids*

Cancer survivors also mentioned uncomfortable and concerning side effects of opioid use as a reason for avoiding use [44, 45, 48, 49]. They reported distressing experiences of being drowsy, "high", numb, constipated, or even more severe side effects such as allergic reactions and breathing difficulties [48]. Others held concerns about the general adverse health effects of opioids and the dangers of using them concurrently with other medications [44, 45]. Participants also described opioid medications as "unnatural" and worried about the effects of having it in their bodies [44].

**Barriers Specific to Physical Activity**

*Logistical Barriers*

Cancer survivors described multiple logistical barriers to engaging in physical activity [43, 52], including interference with work, as well as family and travel plans [43]. One study found that 10.8% of participants cited cost as a barrier to physical activity, 13.5% reported a lack of access to training facilities or equipment, and 10.8% did not have access to knowledgeable exercise staff [52]. Inconvenient commutes or conflicting class times also prevented participants from engaging in formalized physical activity [43].

*Symptom Barriers*

Several symptom-related barriers hindered cancer survivors' ability to regularly engage in physical activity. Pain and fatigue were commonly reported symptoms [42, 43, 45, 46], with one study reporting that 62.2% of cancer survivors endorsed adverse effects from treatment as a barrier to physical activity, and 29.7% experienced limitations from other health issues [52]. Among participants who were able to practice some form of physical activity, exercise often exacerbated pain and fatigue symptoms, which hindered their ability to continue those routines [42, 45]. Cancer survivors' abilities to engage in physical activity were also inhibited by other concurrent physical symptoms, including limited range of motion and neuropathy [43].

*Cognitive Barriers*

Cognitive barriers, mental processes that inhibit the interpretation and application of information, also prevented cancer survivors from engaging in physical activity [43, 52]. A theme that emerged from one of the studies was the impact of negative self-perceptions, which resulted in cancer survivors focusing more on their disabilities rather than their abilities to be actively involved in their pain self-management [43]. Participants also described problems with remaining focused on practicing physical activity [43]. In line with these findings, one study reported that 37.8% of cancer survivors endorsed a lack of self-discipline as a barrier to physical activity [52].

**Barriers Specific to Alternative Pain Management Modalities**

*Financial Barriers*

The most prominent barrier that cancer survivors experienced in pursuing nontraditional pain management modalities was financial [9, 44, 45]. Lack of insurance coverage and resultant high out-of-pocket costs made it difficult for participants to access and/or continue alternative pain management options such as acupuncture [44], massage [9, 44], and cannabis-use [45]. Due to these barriers, cancer survivors felt that they had few accessible alternatives to opioid-mediated pain management [9].

**II. Relationships Within and Between Studies (Concept Mapping)**

Using the findings from the preliminary synthesis, relationships were analyzed through concept mapping. This integrative approach links several pieces of evidence extracted from separate studies in the review to create a model displaying key concepts and depicting their relationships to one another [56].

**Concept Map of Chronic Cancer Pain Management**

The antecedents (triangle), pain management modalities (oval), barriers (rectangle), and consequences (diamond) of effective chronic pain management in cancer survivors are mapped in Figure 1. Effective chronic cancer pain management is the central concept, consisting of opioid analgesic therapy [9, 44, 45, 49, 50], physical activity [9, 43, 45, 47-49, 52], and alternative therapy modalities [9, 44, 45]. Intensive curative treatment, complex pain syndromes, and symptom-related pain are the main factors that predispose individuals to chronic cancer pain [9, 10], which is the primary antecedent required for effective pain management to occur.

## Discussion

Following this review, the most significant patient-related barriers to effective chronic cancer pain management were identified in the strongest of evidence. First, negative perceptions toward chronic pain were reported as a barrier to chronic cancer pain management by five studies, with one high-quality article (level 5 evidence) [42] and four moderate-quality articles (level 6 evidence) [46-49]. Second, patient-provider communication barriers to chronic cancer pain management were mentioned by seven studies, with one high-quality article (level 5 evidence) [42] and six moderate-quality articles (level 6 evidence) [9, 44, 46-49]. Third, opioid-related stigma was endorsed by five moderate-quality articles (level 6 evidence) [9, 44, 45, 48, 50]. Undesirable side effects of opioids were reported in four moderate-quality articles (level 6 evidence) [44, 45, 48, 49]. Fourth, logistical and cognitive-related barriers to physical activity were reported in two high-quality articles (levels 4 and 5 evidence) [43, 52]. Fifth, symptom-related barriers to physical activity were reported in five studies, with two high-quality articles (levels 4 and 5 evidence) [43, 52] and three moderate-quality evidence articles (level 6 evidence) [45, 47, 48].

*Negative Perceptions Toward Chronic Pain and Patient-Provider Communication Barriers*

Consistent with the findings from this review, studies show that patients' perceptions of their illnesses have implications on their health outcomes and behaviors [57, 58]. To facilitate realistic expectations of chronic pain with patients and empathetic conversations about their pain experiences, clinicians should receive training on effective communication strategies and the biopsychosocial nature of pain. Communication enhancement interventions in healthcare have been shown to improve patient satisfaction and health-related decision-making [59, 60]. Biopsychosocial pain education for health care professionals has also been shown to result in improved knowledge and attitudes towards pain and an increased likelihood of adhering to evidence-based practice [61]. These interventions can help providers reassure cancer survivors by understanding their distressing experiences of chronic pain, practicing active listening techniques, and asking open-ended questions [47].

To clarify the roles of pain management team members, chronic cancer pain management would be best conducted by multidisciplinary care teams with designated pain management providers [62]. These teams should consist of not only oncologists and pain specialists, but also social workers, psychologists, and other care providers to holistically consider the biopsychosocial domains of pain [63, 64]. Each clinician's role in pain management should be clearly established to set expectations and structure [65], and members of the team should communicate frequently on a shared platform while providing patient updates and managing transitions in care to enable highly adaptable and centralized communication [66]. Doing so would also address cancer survivors' concerns about being unprepared for chronic pain and receiving inadequate pain-related information, which is supported by a study finding that 30-50% of cancer patients are not informed about common pain symptoms [67]. Due to the logistical challenges of coordinating large care teams, engaging with certain clinicians virtually may be a more accessible approach. Using telehealth technology for chronic pain management has proven to be feasible for both patients and health care professionals [68].

*Opioid Stigma and Undesirable Side Effects*

Misunderstandings about substance use disorders and other concepts related to opioid use are not limited to cancer survivors and have been evidenced by healthcare providers as well [44, 69]. Some clinicians incorrectly interpret tolerance as an indicator of a use disorder, which may limit their opioid prescribing or result in abrupt tapering practices [44, 69]. Although many providers are aware of the need to properly discuss the risks and benefits of opioid usage, as well as alternative options, they continue to feel uncomfortable or unprepared to discuss addiction-related concepts in the context of pain management [44, 70]. This gap can be attributed to factors such as a lack of standardized guidelines for delivering opioid-related information and limited training on how to deliver patient education on opioid use and conduct screening for substance use disorders [44], as well as inadequate training about opioid-related prescribing and opioid use disorder among curricula in both nursing and medical programs [71-73].

To address these needs, healthcare providers require comprehensive education on long-term opioid pain management. Although education interventions for decreasing opioid prescribing have been explored [72, 74], there remains a need for interventions that better equip providers to deliver opioid education to cancer survivors. These interventions should include how to provide patient education on opioid use, normalize conversations about pain, and when to refer cancer survivors to other specialists [44, 75]. Clinicians should also conduct frequent and thorough screening on pain, function, and possible misuse of medication [44, 75].

Educational resources on opioid-mediated pain management and opioid use disorder could be developed for cancer patients and survivors with chronic pain, as addressing negative perceptions of the consequences associated with opioids is pivotal for promoting medication adherence [76]. These resources should include guidelines about storing, handling, and disposing prescription opioids, as well as explanations of the processes of opioid use disorder, tolerance, and overdose [44, 50]. As family and caregivers often play a large role in perceptions of about opioid use [9, 50], they should be included in educational interventions and co-developing care plans.

*Logistical, Symptom-Related, and Cognitive Barriers to Physical Activity*

Engagement in physical activity can be promoted through education and support programs [77]. Cancer survivors should be educated on safe forms of physical activity and included in the conversation of developing recovery plans to gradually improve physical functioning and manage pain [43]. Studies among cancer survivors have reported a desire for health care providers who are actively involved in planning physical activity regimens [43]. Healthcare providers should encourage the use of low-intensity exercises as a foundation to eventually progress to more active physical training [78]. To address anxiety, fear of exacerbating pain, and avoidance behavior, patient education should also include pain monitoring and understanding how to distinguish between normal and concerning levels of pain [43].

Social support interventions can also be leveraged in various settings to promote physical activity for cancer survivors. Studies have found significant correlations between higher levels of social support and likelihood of engaging in physical activity [79, 80]. Peer support groups can provide a supportive environment for cancer survivors to discuss chronic cancer pain experiences, develop meaningful relationships, and find opportunities to engage in physical activity in community [81, 82]. Cancer survivors, providers, and caregivers should be educated on the availability and accessibility of these programs [82].

*Gaps and Future Research*

Based upon this synthesis of the evidence, there were several notable gaps in the cancer survivorship literature on chronic pain management. Behavioral interventions, such as cognitive behavioral therapy, have long been established as an effective primary therapy for chronic pain management [83, 84]. However, while the efficacy of behavioral interventions for chronic pain has been observed in early-stage cancer patients [62, 85], few studies have explored their effectiveness in cancer survivors post-treatment and cancer patients with end-stage disease [62, 86]. Additionally, of the studies that mentioned physical activity, none of them explicitly assessed the usage of formal physical rehabilitation programs. One study did find that 49% of participants (ranging throughout the cancer treatment trajectory), were open to participating in a proposed hybrid exercise program, but the program itself had yet to be implemented [52]. As opioid prescribing policies continue to evolve, future research should also seek to examine their impact on public perceptions, opioid-related stigma, and provider competency in managing chronic cancer pain [9, 44].

#### Strengths and Limitations

This review is one of the first to assess the literature on patient-related barriers to chronic pain management among cancer survivors, and there are several strengths to this novel review. The inclusion of a large variety of study designs, along with a thorough approach to thematic analysis, facilitated a deeper understanding of the unique barriers that cancer survivors face to chronic pain management. This review followed a rigorous approach in accordance with PRISMA and SWiM reporting guidelines [31, 32] and utilized a variety of databases to capture a broad range of studies which were critically appraised by two independent reviewers.

There are also limitations to acknowledge. Across all the included studies, most of the participants were white, female, and had a diagnosis of breast cancer. Additionally, as most of the studies were qualitative in nature, the sample sizes were generally small. These factors limit the generalizability of these findings to the broader cancer survivor population including survivors of other ethnicities, genders, and with different cancer diagnoses.

A few factors may have affected the internal validity of this review. Recall bias was a recurring concern, as findings were completely based on participants' personal recollection of their chronic cancer pain management, which may have occurred many months or years in the past. None of the studies considered the role of sociodemographic factors, such as race, ethnicity, education level, and healthcare insurance coverage, in impacting inequalities in cancer survivors' experiences of pain management barriers. These factors may have further confounded the results within the studies.

### Conclusions

In this systematic review, patient-related barriers to effective chronic pain management among cancer survivors were identified and described. The most significant barriers that emerged were negative perceptions toward chronic pain, inadequate patient-provider communication, opioid-related stigma, undesirable side effects of opioids, and symptom-related barriers to physical activity. To address these barriers, educational interventions should be implemented for healthcare providers focusing on communication techniques, the biopsychosocial nature of pain, opioid use disorder, updated opioid prescribing guidelines, and techniques for practicing effective patient education on chronic cancer pain management. Social support interventions should also be implemented to promote engagement in physical activity. Future studies should investigate the efficacy of these interventions as well as behavioral techniques such as cognitive behavioral therapy, coping skills training, and relaxation with imagery in cancer survivors with chronic pain.

## Declarations

#### Funding

Jonathan Zhu is supported by the following grant: NIH Individualized Care for At Risk Older Adults (T32NR009356).

Connie Ulrich is partially supported by a grant from the NIH Fogarty International Center (D43TW011809) and the Agency for Healthcare Research and Quality grant (R01HS028427).

Peggy Compton is supported by a grant from the Rita and Alex Hillman Foundation.

Jie Deng is receiving research funding support from the Patient-Centered Outcomes Research Institute (IHS-2020C3-21101) and the Department of Defense (HT9425-23-1-0705).

#### Competing Interests

All authors declare they have no relevant financial or non-financial interests to disclose.

#### Author Contributions

J.Z. and P.C. contributed to the review conception and design. The literature search, data analysis, and tables and figures were completed by J.Z. All authors contributed to the drafting and critical revision of the final manuscript.

## Tables

#### Table 1. Quality Appraisal and Level of Evidence of Articles

| Citation | JBI Critical Appraisal Tool Used [38] | Quality Appraisal Score | Level of Evidence [53] |
|---|---|---|---|
| *Armoogum, J., Harcourt, D., Foster, C., Llewellyn, A., & McCabe, C. S. (2020). The experience of persistent pain in adult cancer survivors: A qualitative evidence synthesis. European journal of cancer care, 29(1), e13192.* | Systematic Reviews and Research Syntheses | 10/11 | 5/7 |
| *Chan, A., Ports, K., Neo, P., Ramalingam, M. B., Lim, A. T., Tan, B., ... & Loh, K. (2022). Barriers and facilitators to exercise among adult cancer survivors in Singapore. Supportive Care in Cancer, 30(6), 4867-4878.* | Analytical Cross-Sectional Studies | 6/8 | 4/7 |
| *Chavez, M. N., Tyson, D. M., Lake, P. W., Gutierrez, A., Sherry, P., Rigg, K. K., ... & Lubrano, B. (2022). 'They say you can get addicted': Exploring factors that fuel the fear of addiction to prescription opioids among cancer survivors. European Journal of Cancer Care, 31(3), e13582.* | Qualitative Research | 7/10 | 6/7 |
| *Filipponi, C., Masiero, M., Mazzoni, D., Chichua, M., Marceglia, S., Ferrucci, R., ... & Pravettoni, G. (2024). The voices of breast cancer survivors with chronic pain: A qualitative thematic analysis of patients' challenges to pain management. Journal of Psychosocial Oncology, 1-25.* | Qualitative Research | 7/10 | 6/7 |
| *Hovind, I. L., Bredal, I. S., & Dihle, A. (2013). Women's experience of acute and chronic pain following breast cancer surgery. Journal of clinical nursing, 22(7-8), 1044-1052.* | Qualitative Research | 7/10 | 6/7 |
| *Marshall, V. K., Chavez, M., Efre, A., Lake, P. W., Rigg, K. K., Lubrano, B., ... & Tyson, D. M. (2023). Barriers to adequate pain control and opioid use among cancer survivors: implications for nursing practice. Cancer nursing, 46(5), 386-393.* | Qualitative Research | 7/10 | 6/7 |
| *O'Regan, A., Fish, L. J., Makarushka, C., Somers, T., Fitzgerald Jones, K., Merlin, J. S., ... & Check, D. (2024). Managing chronic pain in cancer survivorship: communication challenges and opportunities as described by cancer survivors. American Journal of Hospice and Palliative Medicine®, 41(1), 78-86.* | Qualitative Research | 7/10 | 6/7 |
| *Peretti-Watel, P., Bendiane, M. K., Spica, L., & Rey, D. (2012). Pain narratives in breast cancer survivors. Pain Research and Treatment, 2012(1), 153060.* | Qualitative Research | 6/10 | 6/7 |
| *Salz, T., Chimonas, S., Jinna, S., Brens, J., Kriplani, A., Salner, A., ... & Korenstein, D. (2024). Pain management for post-treatment survivors of complex cancers: a qualitative study of opioids and cannabis. Pain Management, 14(2), 40-53.* | Qualitative Research | 7/10 | 6/7 |
| *Tyson, D. M., Chavez, M. N., Lake, P., Gutierrez, A., Sherry, P., Rigg, K. K., ... & Pabbathi, S. (2021). Perceptions of prescription opioid medication within the context of cancer survivorship and the opioid epidemic. Journal of Cancer Survivorship, 1-12.* | Qualitative Research | 7/10 | 6/7 |
| *Van Dijck, S., De Groef, A., Kothari, J., Dams, L., Haenen, V., Roussel, N., & Meeus, M. (2023). Barriers and facilitators to physical activity in cancer survivors with pain: a systematic review. Supportive Care in Cancer, 31(11), 668.* | Systematic Reviews and Research Syntheses | 10/11 | 5/7 |

**Table 2. Table of Evidence**

| Citation | Research Objective/Question | Sample | Summary of Results | Implications |
|---|---|---|---|---|
| Armoogum et al. (2020) | What is the experience of persistent pain in adult cancer survivors? | 4 Studies<br><br>- 3 from Scandinavia, 1 from France<br><br>n = 52 Female Breast Cancer Survivors<br><br>Age Range: 26-83 Years (Median: 54.5) | Lack of preparedness and support for persistent pain.<br><br>Physical impact of persistent pain<br><br>Emotional experience of persistent pain<br><br>Conceptualization of persistent pain | - Cancer survivors often feel alone in managing their pain<br><br>- all-pervading nature of pain has a physical impact on their daily lives<br><br>- Cancer survivors need more information about the risks of persistent pain after cancer treatment and support services<br><br>- Healthcare professionals need to operate with an awareness of emotional and existential pain-related distress, rather than lack of empathy |
| Chan et al. (2022) | What are the barriers and facilitators to exercise among Singaporean cancer survivors who are undergoing, or have undergone, chemotherapy at the National Cancer Center, Singapore, and what are their exercise behaviors across the survivorship continuum? | n = 37 (participants who completed chemotherapy)<br><br>18 male, 19 female<br><br>age: 55.8 (mean) ± 12.9<br><br>Cancer Type: Lower GI (7), Breast (7), Hematologic Malignancies (7), Female Reproductive Organs (4), Hepatobiliary System (3), Upper GI (3), Thorax (2), Genitourinary (3), Soft Tissue Sarcoma (1)<br><br>Time Since Chemotherapy Completion: 4.6 ± 3.9 (0-12) | Among participants who had completed chemotherapy, 75.7% met exercise guidelines<br><br>Personal Barriers: Adverse effects from treatment (62.2%), Lack of self-discipline (37.8%), Exercise limited by other health issues (29.7%)<br><br>Social Barriers: Lack of company (29.7%)<br><br>Environmental Barriers: Weather (45.9%), Cost (10.8%), Lack of access to training | - Lack of education among Singaporean cancer survivors about the benefits of exercise<br><br>- Oncologists have a significant burden to be available for extended periods of time and to be knowledgeable regarding exercise and cancer survivorship, and multidisciplinary team support would help to alleviate this burden |

| | | | | |
|---|---|---|---|---|
| | | | facility or equipment (13.5%), Lack of appropriate exercise facility (10.8%), Lack of knowledgeable exercise staff (10.8%) | - Standardized care pathways and implementation plans should be developed to integrate exercise into standard oncology care |
| Chavez et al. (2022) | What are the perceptions and identifying factors that contribute to negative attitudes and fear of opioids among cancer survivors? | n = 25<br>- 24 (96%) female<br>- 13 (52%) Black, 9 White, 3 Hispanic<br>- mean age at diagnosis: 50.84<br>- mean survivorship: 3.92 years from treatment<br>- 22 breast cancer, 3 other | - Cancer survivors' view opioids as an illicit drug<br>- Media narrative of the opioid epidemic exacerbated negative perceptions of opioid use<br>- Experiences of friends and family with OUD informed perceptions of opioids<br>- Poor understanding of terminology resulted in misconceptions about opioid use and addiction<br>- Fear of addiction resulted in unrelieved cancer pain, which then resulted in poor quality of life | - Fear is often formed through personal relationships with family or friends<br>- Addiction disrupts social dynamics, and results in behavioral implications, as well as stealing of medication<br>- Cancer survivors need more information and education around prescription opioid medications<br>- Cancer survivors' fear of addiction overshadows the need to take prescribed opioids<br>- Popular media and news outlets should be used to assist in reducing stigma<br>- Cancer communities and survivorship support groups can incorporate educational opportunities |

| | | | | |
|---|---|---|---|---|
| Filipponi et al. (2024) | What are the unique needs and obstacles related to pain management in Breast Cancer Survivors with Chronic Pain? | n = 17<br><br>all female breast cancer survivors<br><br>average age = 51<br><br>average time after end of radiotherapy/chemotherapy = 7 years (range: 2-16) | 1. Challenges to Pain Management<br><br>Doctor-Patient Communications Barriers<br><br>- doctors downplaying pain intensity<br><br>- physicians primarily viewing pain as a physical sensation<br><br>- lack of empathy from doctors<br><br>Contextual and Societal Barriers<br><br>- issues accessing pain management services: lack of information, limited awareness, absence of practical tools<br><br>2. Self-Management Needs<br><br>- Psycho-social Support<br><br>- Care-related Needs<br><br>- Shared Decision-Making<br><br>3. Treatment Preferences and Perceptions of Pain Management<br><br>- Treatment Preferences: discussion of pharmacological vs. integrative treatments<br><br>- Institution Preference: preference for multidisciplinary centers that can provide personalized interventions<br><br>- Decision Role Perception: participants described experiencing collaborative decision-making, active information seeking, and passive | - Tailored support systems that address patient hesitancy, pain normalization, and healthcare providers' attitudes<br><br>- Informing patients about available pain management services, tools, and treatment options<br><br>- Integrating caregiver and peer support: survivor support groups<br><br>- Refining healthcare provider education<br><br>- Adopting comprehensive multidisciplinary approach to pain management: social workers, psychologists, and other healthcare providers<br><br>- Incorporating eHealth tools: facilitate transaction and continuity of care from hospital to home |

| | | | | |
|---|---|---|---|---|
| | | | acceptance of medical decisions | |
| Hovind et al. (2013) | To gain knowledge of how women experience pain and pain treatment after breast cancer surgery and to identify areas of pain management that they believe could be improved | n = 8<br><br>- all female<br><br>- mean age: 55 | Descriptions of Acute and Chronic Pain<br><br>- all expected acute pain, but none of them had expected chronic pain after months and years<br><br>- words other than pain were used to characterize some of their aching (prickly, hurts, tender)<br><br>Experience of Information<br><br>- many participants were not concerned about any information other than prognosis and surgery during the acute phase<br><br>- need for more information about what should be considered normal, such as the development of persistent pain<br><br>- although many were satisfied with follow-up phone calls from the hospital, they also desired more frequent follow-up visits<br><br>- pain was not emphasized by health professionals even if it was brought up<br><br>Responsibility for Their Own Pain Treatment<br><br>- skepticism about using several types of analgesics, many had previously experienced severe side effects of drugs (allergies and breathing difficulties)<br><br>- many found their own strategies for pain management due to lack of support | Pain Experience After Surgery<br><br>- chronic pain is often unexpected<br><br>Responsibility for Own Postoperative Pain Management<br><br>- often didn't follow recommended analgesic treatment due to fear of addiction and drowsiness<br><br>Need for More Information about Postoperative Pain Management<br><br>- breast cancer patients need to be given more information about pain and pain management post-op so that they can develop a healthy regimen at home<br><br>- phone calls and home visits should be utilized for follow-up |

| | | | | |
|---|---|---|---|---|
| | | | Living with Chronic Pain in Daily Life<br><br>- pain negatively affected work, physical activities, social life, and sleep<br><br>- had to accept living with pain | |
| Marshall et al. (2023) | To explore barriers to pain management and perceptions of opioid use among cancer survivors | 25 Cancer Survivors<br><br>- 24 (96%) female<br><br>- 13 (52%) Black, 9 White, 3 Hispanic<br><br>- mean age at diagnosis: 50.84<br><br>- mean survivorship: 3.92 years from treatment<br><br>- 22 breast cancer, 3 other | 1. Taking Just Enough to Take the Edge Off<br><br>- self-medication behaviors and nonadherence to prescribed regimen<br><br>- participants preferred to endure the pain than risk addiction to the medications prescribed<br><br>- taking medications as needed, less than prescribed, and abruptly discontinuing led to more pain, physical, and psychological distress<br><br>- family and caregiver perceptions often played a significant role in medication-taking behavior<br><br>2. Lack of Insurance Coverage and Costly Alternative Pain Treatment Options<br><br>- non-opioid alternatives were used in some cases (OTC medications, herbs, vitamin supplements, essential oils, hot/cold compresses, warm baths, ROM and stretching exercises, and meditation)<br><br>- lack of health insurance reimbursement was a barrier for alternative therapies | - Improving access to multimodal pain management options and addressing continued education on opioids for pain management (ex: mandating insurance coverage for acupuncture and improving education requirements for HCPs)<br><br>- Improved communication and education between nurses/providers and patients about opioid prescribing, dispensing, and treatment guidelines<br><br>- Addressing concerns related to fears of addiction<br><br>- Clear guidelines for follow-up care of survivors using appropriate terminology and standardized pain assessment tools to elicit patients' experience with chronic pain including severity, duration, aggravating and alleviating factors, interference with daily activities, and patterns |

| | | | | |
|---|---|---|---|---|
| | | | 3. Chronicity of Cancer-Related Pain Not Adequately Addressed and Often Mismanaged<br><br>- providers often encouraged OTC analgesic use, even when pain was not well-controlled<br><br>- patients felt like their pain was misunderstood and pain management specialist referrals often took several weeks<br><br>- questions about validity of pain left patients frustrated with providers<br><br>- pain management was often reactive instead of proactive<br><br>- provider attitudes made them feel like they were drug-seeking | |
| O’Regan et al. (2024) | To better understand cancer survivors' pain management experiences after curative-intent treatment | n = 13<br><br>Cancer Survivors<br><br>- mean age: 57 years<br><br>- 10/13 white<br><br>- 10/13 female<br><br>- 9/13 breast cancer<br><br>- 10/13 private health insurance | Chronic Pain Characteristics and Experiences<br><br>- no longer being able to complete ADLs and some had issues with basic functions such as eating, walking, or sleeping<br><br>- intensity and duration of pain, along with lack of effective remedies led to distress, anxiety, and depression for some<br><br>Cancer Survivor-Provider Interactions<br><br>- importance of feeling understood by care team (primary care, oncologists, palliative care providers, neurologists, and pain specialists)<br><br>- few identified a specific provider as their primary pain provider | - Pain assessment should be conducted in a more comprehensive manner, focusing on the impact of pain on daily functioning<br><br>- Routine assessment is key to foster effective communication about pain between patients and providers<br><br>- Self-management interventions improve patient involvement and understanding of the pain management process<br><br>- Proactive planning to identify |

| | | | | |
|---|---|---|---|---|
| | | | Barriers to Effective Communication About Pain<br><br>1. Cancer Survivors May Minimize Their Pain<br><br>2. When Cancer Survivors do communicate their pain, providers may not adequately acknowledge or validate the pain experience<br><br>Potential Strategies for Improved Communication<br><br>- normalizing pain, asking about it more regularly and in a more open-ended way<br><br>- listening and asking open-ended questions | which providers should be primarily responsible for pain management |
| Peretti-Watel et al. (2012) | 1. Document breast cancer survivors' experiences of chronic pain and how it affected their everyday lives<br><br>2. Explore respondents' attitudes toward pain, especially in terms of how they gave meaning to it and how they dealt with it in their daily lives | n = 21<br><br>Breast Cancer Survivors<br><br>- mean age: 49.76 (range: 26 -83) | 1. Chronic Pain<br><br>- 10/21 participants reported experiencing daily chronic pain<br><br>- pain was breast cancer treatment-related (post-op complications and hormonotherapy side-effects)<br><br>- only 5 were taking painkillers<br><br>- pain handicapped several participants from performing ADLs<br><br>- discrepancy between WHOQOL-BREF survey responses and interview responses in terms of quality of life and ability to perform daily tasks<br><br>2. The Various Meanings of Pain<br><br>- some viewed pain as a necessary ordeal in | - Iatrogenic pain is often endured by cancer survivors and neglected by healthcare professionals<br><br>- Normalization of pain prevents effective pain management from being performed<br><br>- Physicians should be involved in better information breast cancer survivors about chronic pain and how to alleviate it<br><br>- Pharmacological pain management and alternative techniques to cope with chronic pain should be facilitated |

| | | | | |
|---|---|---|---|---|
| | | | order to become cured (justified absence of painkillers)<br><br>- others feel no reason to suffer more since they were already cured of their cancer<br><br>- some did not want to take painkillers if they viewed pain as a permanent condition, since they didn't want to take them for the rest of their lives<br><br>3. Dealing with Pain in Daily Life<br><br>- psychological adaptation was often based on relativization, or comparison with others who were less fortunate than them<br><br>- giving up domestic or leisure activities because of pain when performing them | |
| Salz et al. (2024) | To understand experiences with opioids and cannabis for post-treatment cancer survivors | n = 25<br><br>24 head and neck cancer, 1 lung cancer<br><br>mostly male (80%) and white (92%)<br><br>post-treatment: 18.3 months (ranged from 7 to 35 months)<br><br>mean age: 55.9 | Impact of Pain<br><br>- complicated essential physical activities (eating, work, exercise)<br><br>- diminished psychological health and relationships<br><br>Experiences with Providers and Pain Treatments<br><br>- mostly positive experiences with pain care<br><br>- mostly treated by oncology doctors and nurses<br><br>- opioids were common (69% previously used, 8% currently using)<br><br>- cannabis relatively common (54% previously used, 35% currently using) | - Provider involvement in cannabis or opioid-mediated therapy is vital<br><br>- Sole focus on physical pain is inadequate for most cancer survivors<br><br>- Multiple dimensions of pain should be assessed, such as psychosocial needs |

Experiences with Opioids

- mostly viewed opioids as highly effective

- also, highly cautious about opioids due to side effects (constipation, nausea, dizziness, mentally incapacitation) and perceived harms (fear of addiction, stigma from providers and pharmacists)

- many participants would only take opioids if there were no other options available for severe pain

Experiences with Cannabis for Pain

- most said cannabis was helpful and provided relief from physical pain

- some preferred cannabis to opioids (better relief and fewer side effects, natural, helped with nausea and appetite, helpful with sleep, relieved anxiety and psychological pain)

- some said cannabis exacerbated anxiety, interfered with sleep, worsened dry mouth

- some were concerned about stigma and that cannabis use might affect their employment

- access problems: expensive cost, lack of provider recommendations

Changes in Pain Management Expectations

| | | | | |
|---|---|---|---|---|
| | | | - some participants shifted their focus to pain management and minimizing side effects after completing treatment<br><br>- trying to manage pain while maintaining function<br><br>- adjusting expectations to live with some pain, won't be able to completely alleviate pain | |
| Tyson et al. (2021) | To explore the perceptions of opioid use and misuse in cancer survivorship within the context of the opioid epidemic | n= 55<br><br>30 health care providers (50% medical doctors, 29.2% nurses)<br><br>25 cancer survivors (96% female, 52% Black, mean age: 56)<br><br>- 76% within 5 years of cancer diagnosis<br><br>- 88% breast cancer<br><br>- 72% received surgery, chemotherapy, and radiation | 1. Fear of Addiction and Living with Poorly Managed Pain<br><br>- concern about negative rhetoric surrounding opioids, fueled by media coverage<br><br>- less than optimal pain management, stopping medication abruptly and suffering from opioid withdrawal<br><br>2. The Importance of Patient/Provider Communication and the Need for Education Around the Use/Handling/Disposal of Prescription Opioid Medication<br><br>- need for education on how to dispose of prescription opioids<br><br>- need for clarification on terms used to describe issues related to opioid use (tolerance, dependency, addiction, and pseudo-addiction)<br><br>- lack of standardized way of delivering information about prescription opioids, and HCPs had limited training on delivering patient education or screening for substance use disorders | Important Recommendations:<br><br>- Frequent screening, patient education, involving families/caregivers to assess comfort, function, and possible misuse, overuse, and diversion of medication<br><br>- Need for more support and education for HCPs in delivering patient education, substance use disorder screening, and prescription opioid medication use, storage, and disposal<br><br>- Advocacy for more insurance coverage on alternative pain therapies |

| | | | | |
|---|---|---|---|---|
| | | | 3. Preference for Non-Opioid Alternatives for Pain Management<br><br>- alternative treatments (acupuncture, yoga, aromatherapy, medical marijuana) perceived as more natural and less harmful than prescription opioids<br><br>- insurance does not always cover alternative therapies, making them expensive options | |
| Van Dijck et al. (2023) | To gain insight into the barriers and facilitators to physical activity in cancer survivors afflicted with pain | 5 Studies<br><br>n = 82 participants<br><br>mostly female (only one male participant)<br><br>- 3 studies focused exclusively on breast cancer survivors<br><br>- mean/median age of included articles: 59-64<br><br>- total age range: 39-87 | Logistical Domain<br><br>Barriers:<br><br>- interference of physical activity with work, family, and travel<br><br>- lack of time<br><br>- difficult accessibility due to commute or timing of classes<br><br>Facilitators:<br><br>- limited space required to practice Qigong<br><br>- videos and online resources convenient for home practice<br><br>Symptoms Domain<br><br>Barriers:<br><br>- health-related issues (pain, limited ROM, fatigue, symptoms of neuropathy)<br><br>- physical activity led to increase in CIPN symptoms<br><br>Facilitators:<br><br>- benefits to physical well-being and mental well-being, relaxation, calmness<br><br>Social Domain | - Pain itself is a huge barrier to physical activity, and is closely associated with additional obstacles such as anxiety, fear, and avoidance behavior<br><br>- Pain Science Education may help cancer survivors better understand their pain and capacity to perform physical activity<br><br>- Physical activity education should be a priority for health care providers in discussions about pain management<br><br>- Low-intensity exercises may be a steppingstone to aerobic exercise or resistance training |

| | |
|---|---|
| | Barriers: lack of community, doubt and fear in social environment<br><br>Facilitators: group classes, community, social support, shared experiences |
| | Knowledge on Physical Activity<br><br>Barriers: limited knowledge of appropriate exercises, limited knowledge on safety<br><br>Facilitators: knowledge of benefits, knowledge on practical approach |

**Table 3.** Thematic Analysis Table

| Theme | Sub-theme | Description | Initial Codes | Articles Mentioning this Theme | Examples |
|---|---|---|---|---|---|
| Types of Chronic Cancer Pain Management | Non-Opioid Analgesics | Non-opioid analgesic medications used for chronic cancer pain management | Over-the-Counter Pain Medications<br><br>Non-Opioids | [9, 45, 48, 50] | *"Over the counter medication such as Tylenol or Advil" [50]*<br><br>*"Non-opioid prescribed medications such as duloxetine and gabapentin"[9]* |
| | Opioid Analgesic Therapy | Opioid analgesic medications used for chronic cancer pain management | Opioid Medications<br><br>Opioid Analgesics | [9, 44, 45, 49, 50] | *"Opioids were a common strategy [for pain management]" [45]*<br><br>*"Effective pain management often… includes opioids" [9]* |
| | Physical Activity | Physical activity or exercise used for chronic cancer pain management | Exercise<br><br>Physical Activity | [9, 43, 45, 47-49] | *"Range of motion or stretching exercises" [9]*<br><br>*"[R]egular physical activity and exercise therapy have pain reducing effects in cancer survivors" [43]* |
| Theme | Sub-theme | Description | Initial Codes | Articles Mentioning this Theme | Examples |
| | Alternative Pain Management Modalities | Chronic cancer pain management practices that fall outside the scope of opioid and non-opioid analgesics, and physical activity | Complementary Pain Therapy<br><br>Alternative Pain Therapies<br><br>Alternative Treatments | [9, 44, 45] | *"A variety of herbs, vitamin supplements, and essential oils were also described to assist in pain relief" [9]*<br><br>*"[Participants] had tried cannabis for pain after treatment completion…" [45]*<br><br>*"[P]reference for nonopioid alternatives* |

| | | | | | |
|---|---|---|---|---|---|
| | | | | | *to pain management such as acupuncture, yoga, essential oils/aromatherapy, and medical marijuana..." [44]* |
| General Barriers to Effective Pain Management | Lack of Preparedness for Chronic Pain | Experiences of unanticipated development of chronic pain during cancer survivorship | Unprepared for the Experience of Persistent Pain<br><br>Unexpected Chronic Pain | [42, 48] | *"The absence of preparedness for persistent was evident as 'some participants clearly lacked information about pain'" [42]*<br><br>*"[N]one of the women had expected chronic, persistent pain after months and years" [48]* |
| **Theme** | **Sub-theme** | **Description** | **Initial Codes** | **Articles Mentioning this Theme** | **Examples** |
| | Negative Perceptions Toward Chronic Pain | Adverse beliefs about the nature of chronic pain and its prognosis | Fatalism<br><br>Stoicism<br><br>Normalization of Pain<br><br>Misconceptions About Pain | [42, 46-49] | *"I supposed that this is how I have to live" [48]* |
| | Patient-Provider Communication | Barriers related to a lack of understanding between patients and providers | Lack of Clarity on Which Providers are Responsible for Pain Management<br><br>Difficulty Communicating About Pain<br><br>Lack of Empathy<br><br>Stigmatizing Attitudes Toward | [9, 42, 44, 46-49] | *"Providers disregarded their pain" [9]*<br><br>*"Cancer survivors noted not wanting to discuss their discomfort… due to fear or concern of not being believed or being labeled as a 'hypochondriac', manipulating, or demanding of opioid therapy" [9]* |

| | | | | | |
|---|---|---|---|---|---|
| | | | Pain<br><br>"Psychiatrization" of Pain<br><br>Decision Role Perception | | |

| Theme | Sub-theme | Description | Initial Codes | Articles Mentioning this Theme | Examples |
|---|---|---|---|---|---|
| Barriers Specific to Opioid Analgesic Therapy | Opioid-Related Stigma | Negative beliefs about the addictive and deadly effects of opioid medications | Addiction Stigma<br><br>Fears of Overdose<br><br>Illicit Drug | [9, 44, 45, 48, 50] | *"Many also expressed fear of addiction" [45]*<br><br>*"Participants expressed concern about the negative rhetoric surrounding opioids" [44]* |
| | Opioid-Related Knowledge Deficits | Gaps in knowledge or inaccurate perceptions about the process of developing an opioid addiction and how to handle opioid medications | Poor Understanding of Opioid Tolerance, Physical Dependence, and Opioid Addiction | [44, 50] | *"Poor understanding of… differences between opioid-tolerance, physical dependence and addiction" [50]*<br><br>*"[The] need for more education/information about POM (prescription opioid medications) as well as how to use/handle and dispose of the medication emerged as salient issues" [44]* |

| Theme | Sub-theme | Description | Initial Codes | Articles Mentioning this Theme | Examples |
|---|---|---|---|---|---|
| | Undesirable Side Effects | Negative short-term consequences related to opioid use | Concerning Side Effects<br><br>Severe Side Effects | [44, 45, 48, 49] | *"Some described physical side effects, like constipation, nausea, and dizziness as well as being mentally incapacitated" [45]* |

| | | | | | |
|---|---|---|---|---|---|
| Barriers Specific to Physical Activity | Logistical Barriers | Obstacles to engaging in physical activity related to availability and accessibility | Time Constraints<br><br>Accessibility Barriers<br><br>Interference with Commitments | [43, 52] | *“[I]nterference of physical activity interventions with other commitment such as work, family, and travel” [43]* |
| | Symptom Barriers | Obstacles to engaging in physical activity related to physical or psychological symptoms of cancer survivorship | Physical Limitations<br><br>Health-Related Issues | [43, 45, 47, 48, 52] | *“Health-related issues often limited the possibility to complete the physical activity intervention” [43]* |
| **Theme** | **Sub-theme** | **Description** | **Initial Codes** | **Articles Mentioning this Theme** | **Examples** |
| | Cognitive Barriers | Obstacles to engaging in physical activity related to communal or interpersonal factors | Negative Emotions<br><br>Motivation<br><br>Discipline<br><br>Mind-Body Disconnect<br><br>Focus | [43, 52] | *“[D]ifficulty staying focused was also identified as a barrier” [43]* |
| Barriers Specific to Alternative Pain Management Modalities | Financial Barriers | Obstacles related to participants’ ability to afford alternative pain management modalities | Lack of Health Insurance Reimbursement and Plans<br><br>Expensive Out-of-Pocket Costs | [9, 44, 45] | *“[T]hey were met with barriers such as lack of health insurance and plans covering these alternative treatments” [9]*<br><br>*“[F]ound therapies like massage helpful but had to discontinue them due to out-of-pocket costs” [9]* |

| | |
|---|---|
| | *“Some feared cannabis use would interfere with employment or be otherwise stigmatizing” [45]*<br><br>*“‘[The CBD] was really expensive.” [45]* |

### Figures

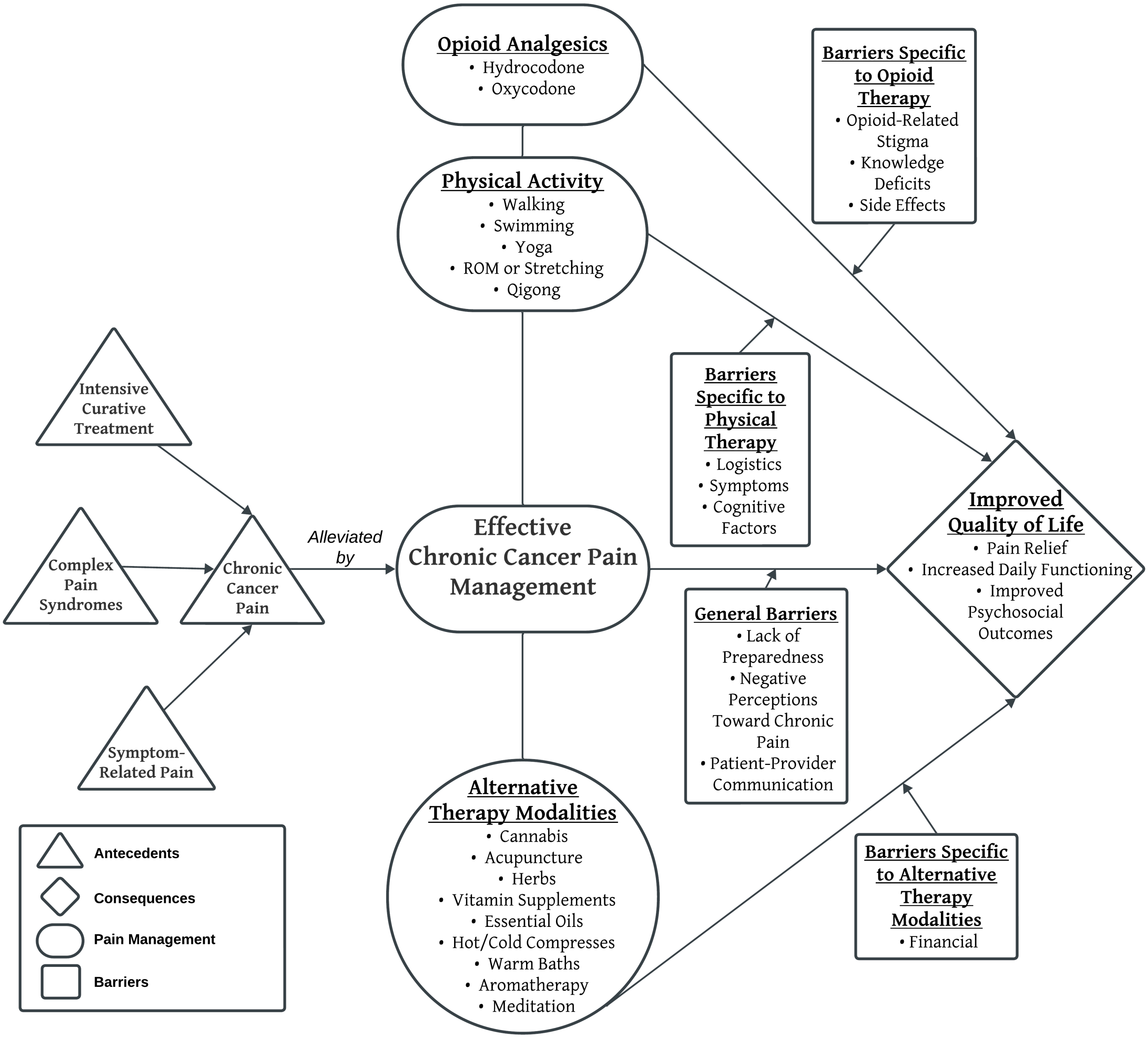

**Figure 1**

**Concept Map of Chronic Cancer Pain Management**

## Supplementary Files

This is a list of supplementary files associated with this preprint. Click to download.

- AppendicesTableofContents.docx
